# Characterization of the First SARS-CoV-2 Isolates from Aotearoa New Zealand as Part of a Rapid Response to the COVID-19 Pandemic

**DOI:** 10.3390/v14020366

**Published:** 2022-02-10

**Authors:** Rhodri Harfoot, Blair Lawley, Leonor C. Hernández, Joanna Kuang, Jenny Grant, Jackson M. Treece, Sharon LeQueux, Robert Day, Susan Jack, Jo-Ann L. Stanton, Mihnea Bostina, James E. Ussher, Miguel E. Quiñones-Mateu

**Affiliations:** 1 Department of Microbiology & Immunology, School of Biomedical Sciences, University of Otago, Dunedin 9016, New Zealand; rhodri.harfoot@otago.ac.nz (R.H.); blair.lawley@otago.ac.nz (B.L.); leonor.hernandez@otago.ac.nz (L.C.H.); kuajo223@student.otago.ac.nz (J.K.); mihnea.bostina@otago.ac.nz (M.B.); james.ussher@otago.ac.nz (J.E.U.); 2 Southern Community Laboratories, Dunedin Hospital, Dunedin 9016, New Zealand; jenny.grant@sclabs.co.nz; 3 Department of Anatomy, School of Biomedical Sciences, University of Otago, Dunedin 9016, New Zealand; jackson.treece@otago.ac.nz (J.M.T.); jo.stanton@otago.ac.nz (J.-A.L.S.); 4 Otago Micro and Nanoscale Imaging, University of Otago, Dunedin 9016, New Zealand; sharon.lequeux@otago.ac.nz; 5 Department of Biochemistry, School of Biomedical Sciences, University of Otago, Dunedin 9016, New Zealand; robert.day@otago.ac.nz; 6 Public Health South, Dunedin 9016, New Zealand; susan.jack@southerndhb.govt.nz; 7 Webster Centre for Infectious Diseases, University of Otago, Dunedin 9016, New Zealand

**Keywords:** SARS-CoV-2, COVID-19, virus isolate, New Zealand, whole-genome sequencing, antiviral

## Abstract

SARS-CoV-2, the virus responsible for the COVID-19 pandemic, has wreaked havoc across the globe for the last two years. More than 300 million cases and over 5 million deaths later, we continue battling the first real pandemic of the 21st century. SARS-CoV-2 spread quickly, reaching most countries within the first half of 2020, and New Zealand was not an exception. Here, we describe the first isolation and characterization of SARS-CoV-2 variants during the initial virus outbreak in New Zealand. Patient-derived nasopharyngeal samples were used to inoculate Vero cells and, three to four days later, a cytopathic effect was observed in seven viral cultures. Viral growth kinetics was characterized using Vero and VeroE6/TMPRSS2 cells. The identity of the viruses was verified by RT-qPCR, Western blot, indirect immunofluorescence assays, and electron microscopy. Whole-genome sequences were analyzed using two different yet complementary deep sequencing platforms (MiSeq/Illumina and Ion PGM™/Ion Torrent™), classifying the viruses as SARS-CoV-2 B.55, B.31, B.1, or B.1.369 based on the Pango Lineage nomenclature. All seven SARS-CoV-2 isolates were susceptible to remdesivir (EC_50_ values from 0.83 to 2.42 µM) and β-D-N^4^-hydroxycytidine (molnupiravir, EC_50_ values from 0.96 to 1.15 µM) but not to favipiravir (>10 µM). Interestingly, four SARS-CoV-2 isolates, carrying the D614G substitution originally associated with increased transmissibility, were more susceptible (2.4-fold) to a commercial monoclonal antibody targeting the spike glycoprotein than the wild-type viruses. Altogether, this seminal work allowed for early access to SARS-CoV-2 isolates in New Zealand, paving the way for numerous clinical and scientific research projects in the country, including the development and validation of diagnostic assays, antiviral strategies, and a national COVID-19 vaccine development program.

## 1. Introduction

It has been two years since the world first learned about the newly discovered SARS-CoV-2, the causative agent of the Coronavirus Disease of 2019 (COVID-19) pandemic [1]. The new respiratory disease was first identified in Wuhan (Hubei province, China) in December 2019, and soon after the whole genome of the novel coronavirus (2019-nCoV or SARS-CoV-2) was sequenced in January 2020 [1]. At that point, it was clear that we were facing another coronavirus outbreak, similar to the zoonotic events caused by SARS-CoV in 2002 and 2003 [2] and MERS-CoV since 2012 [3]. SARS-CoV-2 infections spread rapidly within China, then in early 2020 travel-associated cases were identified in Thailand and Japan, followed by cases in multiple other countries in Asia, Europe and North America [1,4,5,6,7,8,9], which triggered a massive effort to monitor the global distribution and evolution of the virus [10,11,12,13]. As of 6 January 2022, close to 6.8 million SARS-CoV-2 whole-genome sequences had been shared via GISAID (https://www.gisaid.org/, accessed on 6 January 2022) [10].

The report of the initial cluster of cases of acute respiratory illness in Wuhan was quickly followed by the isolation of the novel coronavirus [1]. Whole-genome sequencing of the virus isolate allowed for the classification of SARS-CoV-2 as a member of the subgenus *Sarbecovirus*, genus *Betacoronavirus*, in the family *Coronaviridae* [1]. Six other coronaviruses, two alphacoronaviruses (HCoV-229E and HcoV-NL63) and four betacoronaviruses (HcoV-OC43, HKU1, SARS-CoV, and MERS-CoV), are known to infect and cause disease in humans [14]. In the case of SARS-CoV-2, and similar to other previously discovered novel viruses (e.g., human immunodeficiency virus [15], Ebola virus [16], and even SARS-CoV [2] and MERS-CoV [3]), having access to the actual viral agent responsible for the newly described disease was vital for the characterization of the novel virus, including but not limited to physical features, structure, growth kinetics, cell tropism, transmissibility, pathogenicity, and virulence [1].

Following the isolation of the original Wuhan-Hu-1 SARS-CoV-2 in China [1], research laboratories around the world raced to isolate the virus from local COVID-19 cases. This simultaneous and—at times—coordinated effort, allowed for the dissemination of virus isolates to research laboratories capable of handling infectious viruses, as well as the rapid sharing of non-infectious material to clinical laboratories, public health agencies, and pharmaceutical or biotech companies. This initial work was key to developing and validating diagnostic assays [17,18,19,20,21,22], the screening of novel or re-purposed drugs as prophylactic and/or treatment strategies [23], and the design and development of numerous COVID-19 vaccine candidates [24] in every corner of the world.

The first individual infected with SARS-CoV-2 in New Zealand was diagnosed on 28 February 2020 and a month later, when there were close to 300 confirmed COVID-19 cases, the country went into full lockdown, supported by the use of face masks while maintaining social distancing [25]. This swift measure eliminated the spread of the virus in the community for more than a year, restricting the infections to occasional COVID-19 cases in the international border quarantine facilities [26]. In August 2021, the SARS-CoV-2 Delta (B.1.617.2) variant was introduced in the community and since then has been responsible for a relatively small number of daily COVID-19 cases. As of 4 January 2022, New Zealand had a total of 14,405 COVID-19 cases and 51 deaths (https://nzcoviddashboard.esr.cri.nz/#!/ accessed on 4 January 2022), with increasing infections associated with the highly transmissible SARS-CoV-2 Omicron (B.1.1.529) variant in the quarantine facilities.

We recently described our experience implementing a molecular diagnostic test on a random-access platform (Hologic Panther Fusion^®^ System, Marlborough, MA, USA), right on time to identify the first SARS-CoV-2 infections in New Zealand’s South Island [22]. This work was initially hindered by the lack of access to key material (i.e., SARS-CoV-2 RNA), difficult to obtain during the early days of the pandemic. Here, we describe the first isolation of SARS-CoV-2 in New Zealand, using the first set of patient-derived samples identified in March 2020. We characterized the phenotype and genotype of the first SARS-CoV-2 isolates in the country, which at the time were key in: (i) the distribution of infectious and non-infectious material to multiple clinical and research laboratories, helping validate additional diagnostic assays early in the pandemic and (ii) opening the door to numerous SARS-CoV-2-related projects in New Zealand, including antiviral strategies and the development of COVID-19 vaccine candidates.

## 2. Materials and Methods

### 2.1. Cells

Vero cells (CCL-81™ ATCC), a gift from Dr. Matloob Husain, University of Otago, were grown in high glucose DMEM (Thermo Fisher Scientific, Waltham, MA, USA) supplemented with 5% fetal bovine serum (FBS, Cellgro Mediatech, Manassas, VA, USA), 100 units/mL of penicillin, and 100 μg/mL of streptomycin (Thermo Fisher Scientific). VeroE6/TMPRSS2 [27] cells were purchased from the Japanese Collection of Research Bioresources Cell Bank (Osaka, Japan) and maintained as described above for Vero cells with the addition of 1 μg/mL of Geneticin™ (Thermo Fisher Scientific).

### 2.2. Clinical Specimens

Nasopharyngeal (NP) swabs were collected from individuals with clinical signs or symptoms of COVID-19 in the South Island, New Zealand between March and April 2020. A sterile swab made from Dacron, rayon, or nylon was used for each sample collection, then placed into 3 mL universal transport medium (UTM, various manufacturers). NP samples were transported to the laboratory at room temperature and tested as soon as possible after collection; otherwise, samples were stored at 2 to 8 °C for up to 72 h. After testing, samples were aliquoted and stored at −80 °C. NP samples and basic demographic information were collected with the understanding and consent of each participant. The study was reviewed and approved by the University of Otago Human Ethics Committee (H21/134).

### 2.3. SARS-CoV-2 Isolation

Material from the original clinical specimens (NP swabs in UTM) were mixed 1:1 with high glucose DMEM (Gibco Thermo Fisher Scientific) supplemented with 5% FBS (Cellgro Mediatech), 100 units/mL of penicillin, 100 μg/mL of streptomycin, and 100 µg/mL gentamycin (Thermo Fisher Scientific). The mixture was passed through a 0.45 µm steriflip filter (Merck Millipore, Burlington, MA, USA) and used to inoculate Vero cells (1 × 10^6^ cells/well) in a 48-well plate (Greiner Bio-One, Kremsmünster, Austria). Cells were monitored daily for cytopathic effect (CPE) for five days, and cell-free supernatant from positive cell cultures was used to inoculate 3 × 10^6^ Vero cells in a T-25 flask (Thermo Fisher Scientific). These initial viral stocks (first serial passage or C1) were titrated by determining tissue culture dose for 50% infectivity (TCID_50_) in triplicate with CPE as the end-point using the Reed and Muench method [28]. SARS-CoV-2 titers were expressed as TCID_50_ per milliliter (TCID_50_/mL).

### 2.4. SARS-CoV-2 Plaque Assay

VeroE6/TMPRSS2 cells (1 × 10^6^ cells/well) were seeded in 6-well plates overnight, then exposed to serial 10-fold dilutions of the C1 viral stocks in infection media (DMEM with 2% FBS, Thermo Fisher Scientific) for one hour. A mixture of warm overlay media (2% PBS, 63% DMEM and 35% low melting point agarose, 2 mL/well) was added and the plates incubated at 37 °C, 5% CO_2_, for 72 h. Agarose plugs were removed and plaques fixed with 0.5% crystal violet in 80% methanol for 20 min to determine viral titers by counting plaques and using the Reed and Muench method [28]. SARS-CoV-2 titers were expressed as plaque-forming units per milliliter (PFU/mL).

### 2.5. Immunofluorescence Assay

Vero cells (1.5 × 10^5^ cells) were grown in 24-well plates (Thermo Fisher Scientific), then infected with the SARS-CoV-2 isolates (C1 viral stocks) for 24 h. Cells were fixed with 1.25% glutaraldehyde (Merck Sigma-Aldrich, Darmstadt, Germany) in phosphate buffered saline (PBS, pH 7.4) for 10 min, washed five times (5 min each) with 200 mM sodium borohydride and once with 1× PBS. Cells were permeabilized in 0.1% Triton X-100 (Merck Sigma-Aldrich) for 10 min, then blocked with blocking buffer (0.5% bovine serum albumin (BSA), 0.01% Tween 20 in PBS (PBS-T)). Cells were incubated with the primary antibody (SARS-CoV-2 Spike S1 Antibody, Rabbit MAb protein, Sino Biological, Beijing, China) in blocking buffer for 24 h at 4 °C, then washed four times (5 min each) with PBS-T prior to incubating with the secondary antibody (Goat anti-Rabbit IgG Cross-Adsorbed Secondary Antibody, Alexa Fluor™ 488, Thermo Fisher Scientific) for one hour at room temperature. Cells were washed four times (5 min each) with PBS-T and images recorded with an Olympus IX71 inverted fluorescence microscope (Olympus, Tokio, Japan) using cellSens software (Olympus).

### 2.6. Western Blot

Aliquots of the SARS-CoV-2 isolates (C1 viral stocks) were mixed 1:1 with radioimmunoprecipitation assay (RIPA) buffer (10 mM NaCl, 1% NP-40, 0.5% sodium deoxycholate, 0.1% sodium dodecyl sulfate (SDS), 50 mM Tris pH 8.0, and 20 mM Tris-HCl pH 7.5), incubated for 10 min at room temperature, then frozen at −80 °C for 24 h. Protein lysates were thawed and mixed with 6× denaturing loading buffer (4% SDS, 5% β-mercaptoethanol, 20% glycerol, 0.004% bromophenol blue, and 0.125 M Tris HCl pH 6.8), heated at 70 °C for 15 min, loaded onto a 8% SDS-polyacrylamide gel topped with a 5% stacking gel (BioRad, Hercules, CA, USA), and run at 120 V for one hour in 1× SDS-PAGE running buffer. Proteins were transferred onto a Immobilon-FL membrane (Merck KGA, Darmstadt, Germany) in 1× transfer buffer (25 mM Tris base, 0.1% SDS, and 10% methanol) overnight at 15 V. Membranes were blocked in 0.5% BSA and 0.01% Tween-20 in PBS for one hour, then incubated with the primary antibody (SARS-CoV-2 Nucleocapsid Antibody, mouse mAb, ProSci Inc, Poway, CA, USA) in blocking solution for 2 h. Membranes were washed (3×) in PBS with 0.1% Tween 20, then incubated for one hour with the secondary antibody (Goat anti-mouse Alexa Fluor™ Plus 800, Thermo Fisher Scientific) diluted 1:10,000 in blocking solution. Membranes were examined and protein bands detected using an Odyssey^®^ XF Imaging System (LI-COR, Lincoln, NE, USA).

### 2.7. SARS-CoV-2 RT-qPCR Assay

Individuals infected with SARS-CoV-2 were diagnosed using a SARS-CoV-2 RT-qPCR assay implemented on the Hologic Panther Fusion^®^ System as described [22]. SARS-CoV-2 in cell-free supernatant was quantified using an in-house assay adapted from Corman et al. [17] as previously described [22].

### 2.8. Whole-Genome Sequencing of Patient-Derived SARS-CoV-2 and SARS-CoV-2 Isolates

Fourteen samples (7 clinical specimens and 7 SARS-CoV-2 isolates) were deep sequenced using two different platforms: MiSeq (Illumina, San Diego, CA, USA) and Ion PGM™ System (Ion Torrent™, Thermo Fisher Scientific). Aliquots of the seven clinical NP samples originally stored at −80 °C and of the seven C1 viral stocks grown in Vero cells (SARS-CoV-2 isolates) were used to extract total RNA (QIAamp Viral RNA mini Kit, QIAGEN, Hilden, Germany) and eluted in 20 µL of DNase/RNase-free water as described [22].

#### 2.8.1. MiSeq, Illumina

Aliquots of the 14 RNA samples were used to synthesize complementary DNA (cDNA). Briefly, RNA samples (4 µL) were incubated with 1 µL of DNAse I (1 U/µL, Promega, Madison, WI, USA) at 37 °C for 30 min, then the reaction was stopped by adding 1 µL of DNase Stop Solution (Promega) and incubated at 65 °C for 10 min. cDNA was randomly synthesized as follows: RNA samples were incubated with adapter appended random nonamer primers (5′-GCCGACTAATGCGTAGTCNNNNNNNNN-3′, 50 pM, IDT) at 85 °C for 2 min and room temperature for 20 min. This mixture was then reverse transcribed in 10 µL reactions containing 0.5 µL of SuperScript™ III (200 U/µL, Thermo Fisher Scientific), 2 µL of 5× First-Strand Buffer (250 mM Tris-HCl, 375 mM KCl, 15 mM MgCl_2_, Thermo Fisher Scientific), 0.5 µL of dNTPs (0.5 µL), 1 µL of DTT (0.1 mM), and 0.5 µL of RiboLock RNase inhibitor (40 U/µL, Thermo Fisher Scientific), then incubated at 25 °C for 10 min, 50 °C for 60 min, 95 °C for 2 min, and 4 °C for 2 min. Second-strand DNA was generated using Sequenase™ Version 2.0 DNA Polymerase (Thermo Fisher Scientific) by ramping the temperature from 4 to 37 °C over a period of 8 min, then incubating at 37 °C for 60 min and 94 °C for 2 min. cDNA/second-strand products were amplified in 25 µL reactions containing 0.5 µL of DreamTaq DNA polymerase (5 U/µL, Thermo Fisher Scientific), 2.5 µL of DreamTaq 10× Buffer, 0.5 µL of dNTPs (10 mM), and 0.5 µL of adapter sequence (100 pM/µL, 5′-GCCGACTAATGCGTAGTC-3′) with the following cycling conditions: one cycle at 95 °C for 2 min, 30 cycles of 95 °C for 30 s, 55 °C for 30 s, and 72 °C for 90 s, and one cycle at 72 °C for 5 min. PCR products (amplicons) were purified (QIAquick PCR Purification Kit, Qiagen), the concentration of double-stranded cDNA (dscDNA) quantified (Qubit 2.0, Thermo Fisher Scientific), and stored at −80 °C until further use. Dual indices (barcodes) and Illumina sequencing adapters were added to the 14 amplicons (1 ng) by indexing PCR products using the Nextera XT DNA Library Preparation kit (Illumina), followed by DNA purification (Agencourt AMPure XP, Beckman Coulter). Individual barcoded DNA samples were then quantified (Qubit 2.0, Thermo Fisher Scientific), normalized to 4 nM and pooled. The paired-end multiplexed library (two samples plus 5% PhiX as internal control) were diluted to 20 pM and denatured with NaOH prior to sequencing on the MiSeq system (Illumina) using the MiSeq Reagent Kit v3 600 cycle (2 × 300 bp, Illumina). Indexed reads were demultiplexed and filtered to remove short reads (<80 bp), generating sample-specific fastq files using BaseSpace (Illumina). Fastq files were analyzed using a combination of software packages to characterize the whole-genome SARS-CoV-2 sequences: (i) GISAID (https://www.gisaid.org/, accessed on 2 June 2020) [10], (ii) DRAGEN Bio-IT Platform (Illumina), (iii) Genome Detective Virus Tool (https://www.genomedetective.com/ accessed on 2 June 2020) [29], and (iv) CZ ID (formerly IDseq, https://czid.org/ accessed on 2 June 2020) [30]. Coronapp (http://giorgilab.unibo.it/coronannotator/ accessed on 2 June 2020) [31] was used to annotate and verify the mutations identified with the DRAGEN Bio-IT Platform, the Genome Detective Virus Tool, and CZ ID.

#### 2.8.2. Ion PGM™ System, Ion Torrent™

Aliquots of the 14 RNA samples (7 clinical specimens and 7 SARS-CoV-2 isolates) were deep sequenced using the Ion AmpliSeq™ SARS-CoV-2 Research Assay, which consists of two 5X primer pair pools targeting 237 amplicons (ranging from 125 to 257 bp) specific to SARS-CoV-2 and 5 human expression controls, with >99% coverage of the SARS-CoV-2 genome. Briefly, RNA was quantified (Qubit™ RNA HS Assay Kit, Thermo Fisher Scientific) and 10 ng was used to synthesize cDNA with the SuperScript™ VILO™ cDNA Synthesis Kit (Thermo Fisher Scientific), incubating at 42 °C for 30 min, 85 °C for 5 min, and 10 °C for 2 min. cDNA samples were amplified with the two primer pools from the Ion AmpliSeq™ SARS-CoV-2 Research panel using the Ion AmpliSeq™ Library Kit 2.0 (Thermo Fisher Scientific) with the following cycling conditions: one cycle at 98 °C for 2 min, 16 cycles of 98 °C for 15 s and 60 °C for 4 min, holding at 10 °C using a GeneAmp™ PCR System 9700 (Thermo Fisher Scientific). The two pools of amplicons for each sample were phosphorylated, and the primers partially digested, with the FuPa reagent and combined using the following cycling conditions: one cycle at 50 °C for 10 min, 55 °C for 10 min, 60 °C for 20 min, and holding at 10 °C for <1 h. The P1 adapter and one of 14 barcodes (Ion Xpress™ Barcode Adapters 1–16 Kit, Thermo Fisher Scientific) were ligated to each sample using the following cycling conditions: one cycle at 22 °C for 30 min, 68 °C for 5 min, and 72 °C for 5 min (storing at −20 °C until further use). Barcoded libraries were thawed, purified (Agencourt AMPure XP, Beckman Coulter, Brea, CA, USA), quantified using the Ion Library TaqMan™ Quantitation Kit (Thermo Fisher Scientific), pooled in equimolar concentrations, and templates prepared and enriched for sequencing using the Ion PGM™ Hi-Q™ View OT2 Kit (Thermo Fisher Scientific) in the Ion OneTouch™ 2 System (Thermo Fisher Scientific). Multiplexed barcoded libraries were loaded into one of three Ion 318™ v2 Chips (Thermo Fisher Scientific) and sequenced on the Ion PGM™ using the Ion PGM™ Hi-Q™ View Sequencing Kit (Thermo Fisher Scientific). Signal processing, base calling, and complete sequence analysis was performed using the Torrent Suite Software 5.12.1 and the SARS-CoV-2 Research Plug-in Package (Thermo Fisher Scientific).

### 2.9. Microbiota Analysis

Sequences obtained with the MiSeq (Illumina) platform were analyzed with CZ ID (https://czid.org/ accessed on 2 June 2020) to detect and quantify pathogens (bacteria and viruses) in the clinical specimens (NP swabs) as well as from the cell-free supernatant from the Vero cell cultures (SARS-CoV-2 isolates).

### 2.10. Phylogenetic Analysis

A small subset of whole-genome sequences was downloaded from the GISAID database (https://www.gisaid.org/ accessed on 2 June 2020) [10] in June 2020 to assess the phylogeny of the SARS-CoV-2 sequences described in this study, i.e., 28 SARS-like betacoronaviruses and 70 contemporary SARS-CoV-2 sequences from different lineages. Whole-genome SARS-CoV-2 consensus sequences, corresponding to each patient-derived NP sample, were aligned using ClustalW [32] and their phylogeny reconstructed using the Maximum Likelihood model with bootstrap as the variance estimation method (1000 replicates) implemented within MEGA 6.1 [33].

### 2.11. Electron Microscopy

Vero cells were infected with the SARS-CoV-2 isolates (C1 viral stocks, multiplicity of infection [MOI] of 1) for three days. Cells were scraped into the medium and fixed 1:1 with 2.5% glutaraldehyde in 0.1 M cacodylate buffer for 10 min at room temperature. Cells were then removed from the Biosafety Level-3 (BSL-3) laboratory and spun down again at 300 rcf for 5 min in a PC2 laboratory. The cell-free supernatant was removed, replaced with fresh fixative solution, and stored at 4 °C overnight. Cell suspensions were thawed and centrifuged at 500 rcf for 5 min. The supernatant was removed and 100 µL of the concentrated cell suspension was mixed with 100 µL of 6% low-melting-point agarose (Merck Sigma-Aldrich) in 0.1 M cacodylate buffer, then warmed to 50 °C for 5 min to merge the agarose with the sample. After cooling, the agarose blocks were cut into small pieces, then washed with 0.1 M cacodylate buffer for 30 min in new glass vials. Samples were stained with 1% OsO_4_ in 0.1 M cacodylate buffer for 60 min and stored in 0.1 M cacodylate buffer at room temperature overnight. Samples were washed in cacodylate buffer for 15 min and double-distilled water for 15 min, then stained in uranyl acetate for 60 min. Samples were dehydrated in a graded ethanol series, i.e., 50% for 10 min, 70% for 10 min, 95% for 10 min, 100% for 15 min, and 100% for 20 min, followed by two washes with propylene oxide for 20 min each. Samples were then infiltrated in a propylene oxide resin mixture with ascending concentration of resin (i.e., 50% for 1 h, 66% ratio for 1 h, and 75% ratio for 1 h), incubated in 100% EMBED 812 epoxy resin (Merck Sigma-Aldrich) with benzyldimethylamine (BMDA, Thermo Fisher Scientific) for 1 h, followed by a resin change and stored overnight at room temperature. Samples were infiltrated with fresh resin for 60 min and cured at 60 °C for three days. Thin sections (100 to 200 nm) were cut using a Leica EM UC6 ultramicrotome (Leica Microsystems, Wetzlar, Germany) equipped with a 45° diamond knife (Diatome, Nidau, Switzerland). Sections were collected on formvar-coated copper grids (Merck Sigma-Aldrich) and stained with 2% uranyl acetate (Merck Sigma-Aldrich), then with 0.02% lead citrate either manually or with the automated EMStain11 (Leica Microsystems). Ultrathin sections (100 nm thick) were examined using a Philips CM 100 BioTWIN transmission electron microscope (Philips/FEI Corporation, Eindhoven, Holland). For electron tomography, 200 nm thin sections were visualized at 200 kV on a JEOL 2200 FS field emission scanning electron microscope (JEOL Ltd., Tokyo, Japan) fitted with a TVIPS F416 CMOS camera (TVIPS, Gauting, Germany). Tilt series were collected using serial EM [34] over an angular range between −70° and +70°, at 1° increments. Tomographic reconstruction was performed using the IMOD software suite [35] using the back projection method and were slightly denoised using an anisotropic nonlinear filter. Surface representations were obtained using Chimera software [36].

### 2.12. Viral Growth Kinetics Analysis

The ability of the seven SARS-CoV-2 isolates (C1 viral stocks) to replicate in Vero or VeroE6/TMPRSS2 cells was determined by measuring viral growth kinetics as described [37,38]. Briefly, 1 × 10^6^ Vero or VeroE6/TMPRSS2 cells (seeded in 6-well plates) were infected in triplicate at an MOI of 0.001 for one hour at 37 °C, 5% CO_2_. SARS-CoV-2-infected cells were then washed two times with 1× PBS, cell culture medium replenished (2.5 mL), and incubated at 37 °C, 5% CO_2_ for 7 days. Cell-free culture supernatant was assayed for up to seven days post-infection. The amount of replication-competent SARS-CoV-2 (TCID_50_ values) was determined by CPE, RT-qPCR assay [22], or a cell protection assay based on the Pierce™ BCA Protein Assay Kit (Thermo Fisher Scientific). Viral replication was quantified using the slope of the growth curves and performing linear regression analysis derived from the equation *log(y) = mt + log(h*), where *y* is virus quantity, *t* is time in days, and *h* is the *y*-intercept (day 0). All slope values for each virus were used to calculate the mean, standard deviation, and 10th and 90th percentiles. Differences in the mean values were evaluated using a One-Way Analysis of Variance test (GraphPad Prism v.9.3.1, GraphPad Software, La Jolla, CA, USA).

### 2.13. Drug Susceptibility

The susceptibility of the seven SARS-CoV-2 isolates (C1 viral stocks) to remdesivir (GS-5734, Sapphire Bioscience, Waterloo, Australia), β-D-N^4^-hydroxycytidine (NHC, EIDD-1931, molnupiravir, Sapphire Bioscience), favipiravir (T-705, Sapphire Bioscience), and a SARS-CoV-2 spike neutralizing monoclonal antibody (mAb, rabbit mAb, cat. 40592-R0004, SinoBiological, Beijing, China) was evaluated in VeroE6/TMPRSS2 cells. Serial dilutions spanning empirically determined ranges of each drug were added in triplicate to 96-well plates containing VeroE6/TMPRSS2 cells (20,000 cells/well) and incubated at 37 °C, 5% CO_2_, for two hours. Cells were then infected with the corresponding SARS-CoV-2 isolate at an MOI of 0.005 IU/cell for one hour at 37 °C, 5% CO_2_. Virus inoculum was removed, cells washed twice and complete medium with the corresponding drug dilution replenished. SARS-CoV-2 replication was quantified 72 h post-infection by CPE, RT-qPCR assay [22], or a cell protection assay based on the Pierce™ BCA Protein Assay Kit (Thermo Fisher Scientific). Drug concentrations required to inhibit SARS-CoV-2 replication by 50% (EC_50_) were calculated by plotting the percent inhibition of virus replication versus log_10_ drug concentration and fitting the inhibition curves to the data using nonlinear regression analysis (GraphPad Prism v.9.3.1, GraphPad Software).

### 2.14. Statistical Analysis

Descriptive results are expressed as median values, standard deviations, and confidence intervals. Pearson’s correlation coefficient was used to determine the strength of association between categorical variables. Group means were compared using a 2-sided *t*-test, and group medians were compared using a 2-sided Wilcoxon–Mann–Whitney test. The cutoff level for significance was set at 0.05 (*p* < 0.05). As described above, differences in the mean of the slope values for the viral growth kinetics curves were determined using a One-Way Analysis of Variance test. All statistical analyses were performed using GraphPad Prism v.9.2.0 (GraphPad Software, San Diego, CA, USA) unless otherwise specified. Whole-genome SARS-CoV-2 sequences obtained by deep sequencing in this study have been submitted to GISAID (https://www.gisaid.org/ accessed on 2 June 2020) under the following accession numbers: EPI_ISL_8802822 (NZ1_patient or hCoV-19/New Zealand/NZ1_patient/2020), EPI_ISL_8802829 (NZ1_virus or hCoV-19/New Zealand/NZ1_virus/2020), EPI_ISL_8802823 (NZ2_patient or hCoV-19/New Zealand/NZ2_patient/2020), EPI_ISL_8802830 (NZ2_virus or hCoV-19/New Zealand/NZ2_virus/2020), EPI_ISL_8802824 (NZ3_patient or hCoV-19/New Zealand/NZ3_patient/2020), EPI_ISL_8802831 (NZ3_virus or hCoV-19/New Zealand/NZ3_virus/2020), EPI_ISL_8802825 (NZ4_patient or hCoV-19/New Zealand/NZ4_patient/2020), EPI_ISL_8802832 (NZ4_virus or hCoV-19/New Zealand/NZ4_virus/2020), EPI_ISL_8802826 (NZ5_patient or hCoV-19/New Zealand/NZ5_patient/2020), EPI_ISL_8802833 (NZ5_virus or hCoV-19/New Zealand/NZ5_virus/2020), EPI_ISL_8802827 (NZ6_patient or hCoV-19/New Zealand/NZ6_patient/2020), EPI_ISL_8802834 (NZ6_virus or hCoV-19/New Zealand/NZ6_virus/2020), EPI_ISL_8802828 (NZ7_patient or hCoV-19/New Zealand/NZ7_patient/2020), and EPI_ISL_8802835 (NZ7_virus or hCoV-19/New Zealand/NZ7_virus/2020).

## 3. Results

### 3.1. First COVID-19 Cases in the South Island, New Zealand

**Figure 1:**
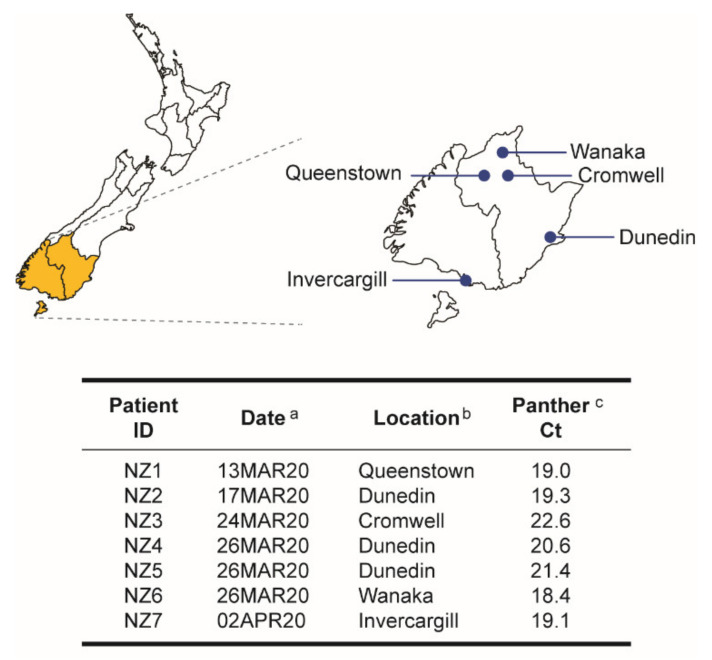
Demographic and clinical characteristics of the study cohort. Nasopharyngeal samples were obtained from seven individuals with clinical signs or symptoms of COVID-19 in New Zealand. Patients were identified (ID) as NZ#. ^a^ Date the clinical sample was obtained. ^b^ City in the South Island, New Zealand, where the sample was collected. ^c^ Cycle threshold (Ct) values of the patient-derived specimens, quantified using the SARS-CoV-2 RT-qPCR assay implemented on the Hologic Panther Fusion^®^ System [22].

In January 2020, the first cases of SARS-CoV-2 were being detected outside China [4,39,40]. In March 2020, during the first four weeks of the SARS-CoV-2 outbreak in New Zealand, close to 200 COVID-19 cases were identified in the Southern District Health Board (DHB) region, most of them detected or confirmed using our then recently implemented RT-qPCR assay on the Hologic Panther Fusion^®^ System [22]. NP samples from seven individuals with clinical symptoms of COVID-19 (termed NZ1 to NZ7) were collected and transported to the BSL-3 laboratory between 13 March and 2 April 2020. Patients were from five locations in the Southern region, age ranging from 16 to 69 years (median 48 years), and had a mean cycle threshold (Ct) value of 20.1 (range 18.4 to 22.6) using the RT-qPCR assay in the Hologic Panther Fusion^®^ System [22] (Figure 1).

### 3.2. First Isolation of SARS-CoV-2 in New Zealand

**Figure 2:**
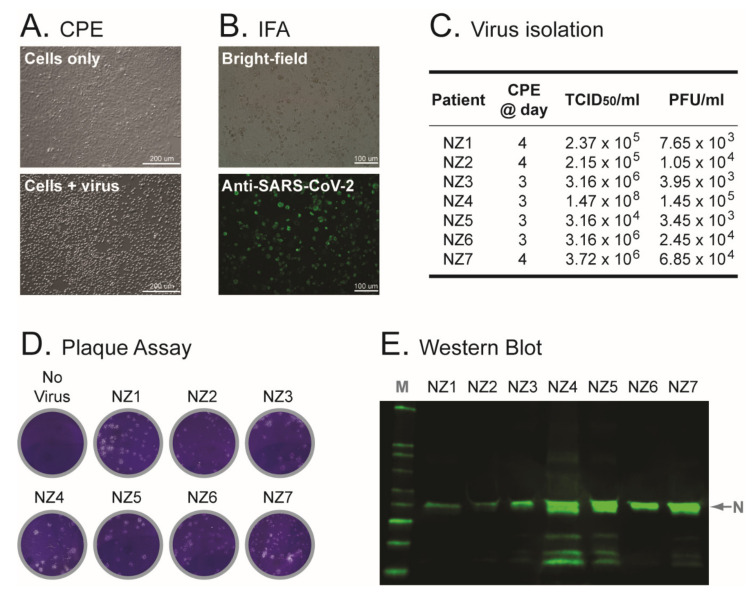
Characterization of the SARS-CoV-2 isolates. (**A**) Viral cytopathic effect (CPE) observed in Vero cells three to four days post-inoculation with the patient-derived nasopharyngeal samples (cells + virus). Cells only, uninfected (control) cells. (**B**) Detection of SARS-CoV-2-infected cells using an immunofluorescence assay (IFA) with a SARS-CoV-2 Spike S1 Antibody, Rabbit MAb protein (Sino Biological) and Alexa Fluor™ 488-conjugated goat anti-Rabbit IgG (Thermo Fisher Scientific). (**C**) Metrics of the seven SARS-CoV-2 isolates. CPE @ day, day post-inoculation with the patient-derived nasopharyngeal samples when the Vero cells first showed viral CPE. Titers of the first serial passage (SARS-CoV-2 stocks) determined by CPE (tissue culture dose for 50% infectivity, TCID_50_) and plaque assay (plaque-forming units per milliliter, PFU/mL). (**D**) SARS-CoV-2 plaque assay using VeroE6/TMPRSS2 cells. No virus, only control cells. (**E**) Western blot of SARS-CoV-2 isolates (C1 viral stocks) using SARS-CoV-2 Nucleocapsid Antibody, mouse mAb (ProSci Inc) and Alexa Fluor™ 488-conjugated goat anti-Rabbit IgG (Thermo Fisher Scientific). Membranes were examined and protein bands detected using the Odyssey^®^ XF Imaging System (LI-COR). M, marker or Western blot protein ladder; N, nucleocapsid.

Material from the original patient-derived samples, i.e., NP swabs in UTM, was used to inoculate Vero cells. All seven cell cultures showed signs of potential virus replication (cytopathic effect) 3 to 4 days post-inoculation, compared with the unexposed (control) cells (Figure 2A). Cell-free supernatant was collected and used to: (i) identify the virus by detecting SARS-CoV-2 E (envelope) and RdRp (RNA-dependent RNA polymerase) genes using RT-qPCR [22] (data not shown); (ii) perform whole-genome sequencing of the virus isolates (see below); and (iii) grow the viruses in a subsequent blind passage in Vero cells (first serial passages or C1 viral stocks). SARS-CoV-2 infection was confirmed by detecting expression of the SARS-CoV-2 spike glycoprotein in the inoculated Vero cells using an indirect immunofluorescence assay (Figure 2B). Viral titers of the first serial passages (C1), determined by infecting Vero cells, ranged from 3.16 × 10^4^ to 1.47 × 10^8^ TCID_50_/mL (median 3.16 × 10^6^ TCID_50_/mL) using CPE as the end-point or 3.45 × 10^3^ to 1.45 × 10^5^ PFU/mL (median 1.05 × 10^4^ PFU/mL) by plaque assay (Figure 2C,D). Vero cells from the first serial passages were also collected, lysed and used to detect the SARS-CoV-2 nucleocapsid protein by Western blot (Figure 2E).

### 3.3. Electron Microscopy of Vero Cells Infected with SARS-CoV-2 Isolates

**Figure 3:**
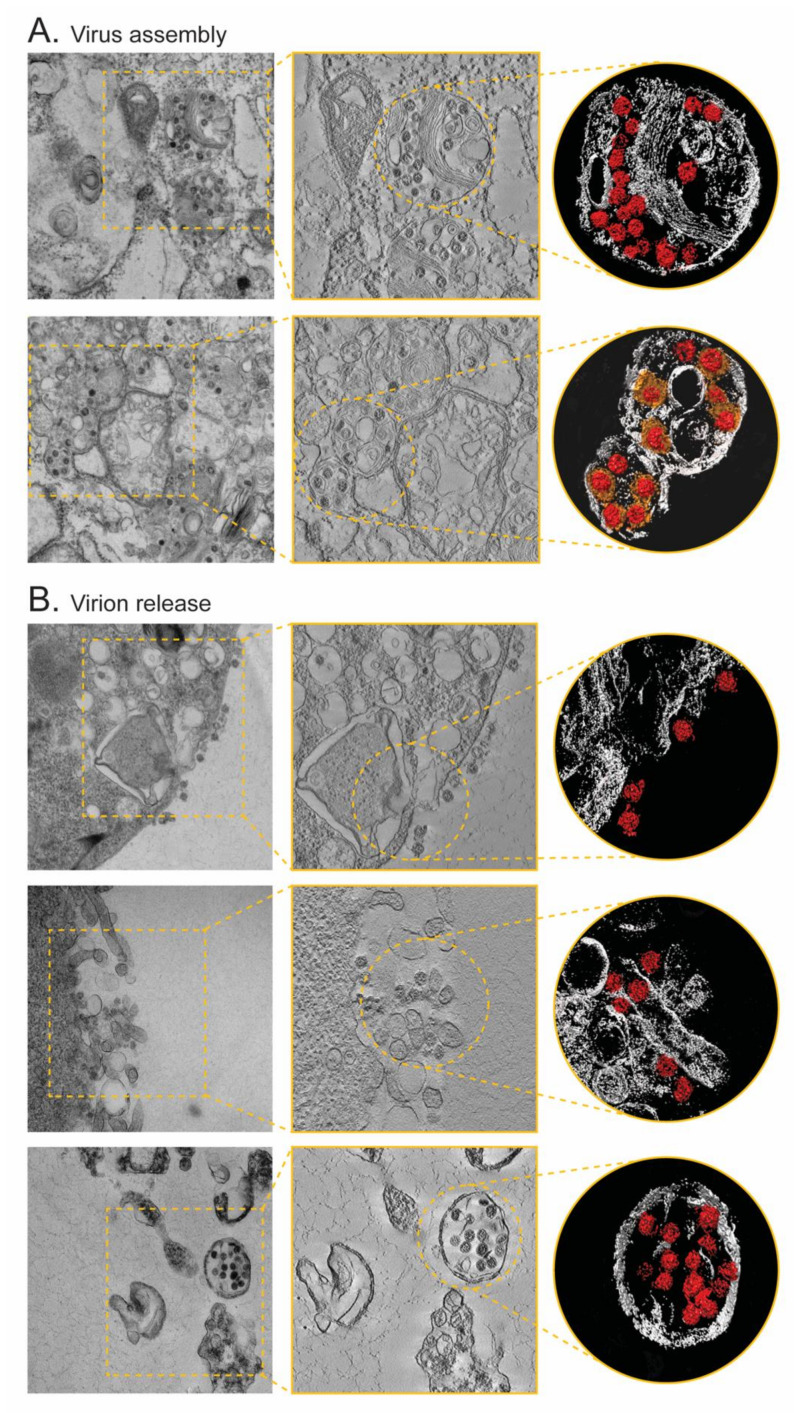
Electron tomography of Vero cells infected with SARS-CoV-2 showing virus assembly sites (**A**) and virion release (**B**). Images (left panels) indicate areas from which regions were selected for tomography. Sections through a tomographic reconstruction (center panels) and surface representations of selected areas (right panels). Virions are rendered in red, while the interior small vesicles wrapped around virions are depicted in orange.

Vero cells from the first serial passages were fixed and resin blocks were sectioned for visualization. Sections 100 nm thick were used for collecting micrographs, while 200 nm sections were used to collect tilt series and perform three-dimensional reconstructions using electron tomography. No differences were observed among the samples prepared from all seven C1 viral stocks, with abundant double-membrane vesicles (diameter of 200 to 400 nm) present in clusters through the cytoplasm in all infected cells (Figure S1). Virions were observed inside enlarged vesicles containing either single or groups of multiple viral particles, usually associated with large membrane stacks that can display either a multi-layered or a concentric arrangement (Figure 3A). Interestingly, numerous “enveloped” virions wrapped by a tight membrane were observed, always enclosed in larger vesicles together with regular virions (Figure 3A and Figure S2). All seven preparations showed newly formed virions being released from infected cells, some still bound to the cellular membrane, and others engulfed in virus-containing vesicles (Figure 3B). Micrographs of 100 nm resin sections showed coronavirus particles as compact, circular, dense structures that, at higher magnification, appear punctuated by an alternation of black and white dots. Electron tomography on thicker sections clearly identified SARS-CoV-2 virions, detecting the characteristic spike glycoproteins in virus particles located in less dense environments such a vesicles or extracellular compartments (Figure S3).

### 3.4. Whole-Genome Sequencing of Patient-Derived SARS-CoV-2 Samples and SARS-CoV-2 Isolates from Early in the Pandemic in New Zealand

**Figure 4:**
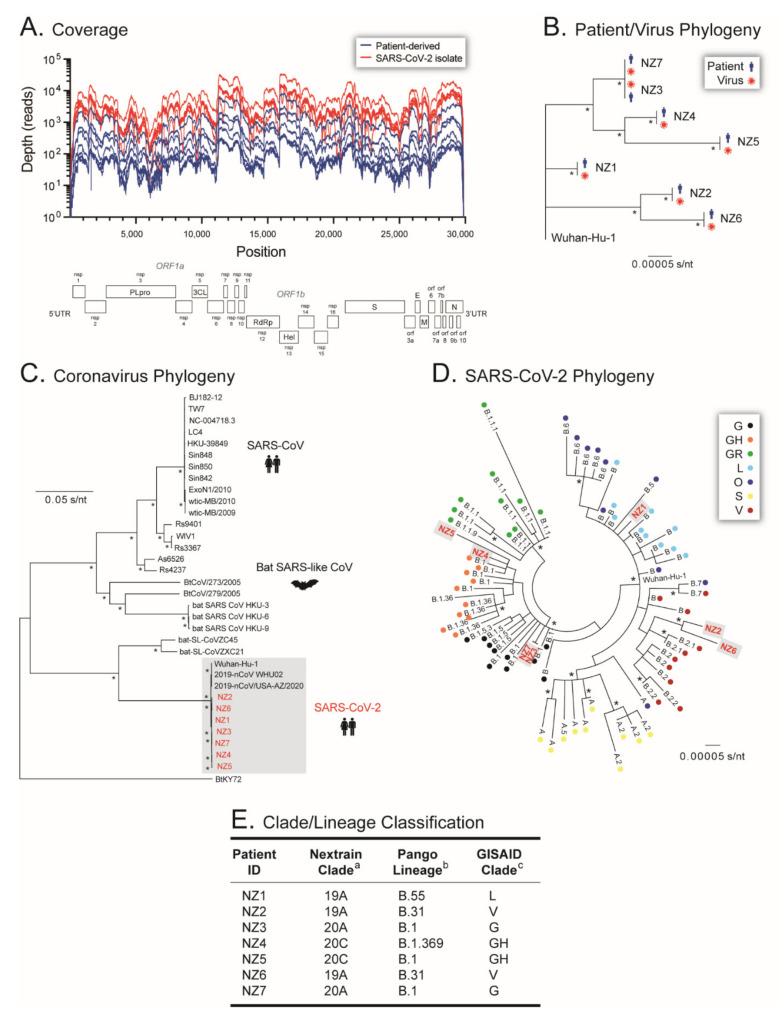
Whole-genome sequencing of SARS-CoV-2 isolates from early in the pandemic in New Zealand. (**A**) Coverage, i.e., number of reads per nucleotide position obtained by deep sequencing the patient-derived samples (blue) and SARS-CoV-2 isolates (red) using the MiSeq platform (Illumina). The position relative to the SARS-CoV-2 isolate Wuhan-Hu-1 NC_045512 is indicated. Maximum Likelihood phylogenetic trees were constructed using (**B**) whole-genome SARS-CoV-2 consensus sequences obtained from all seven patient-derived samples and the seven SARS-CoV-2 isolates, rooted with the Wuhan-Hu-1 NC_045512 sequence, (**C**) whole-genome consensus sequences of the seven SARS-CoV-2 isolates and 28 SARS-like betacoronaviruses, and (**D**) whole-genome consensus sequences of the seven SARS-CoV-2 isolates and 70 contemporary SARS-CoV-2 sequences from different lineages obtained from GISAID database (https://www.gisaid.org/, accessed on 6 June 2020). Each color-coded dot represents SARS-CoV-2 GISAID clades. Bootstrap resampling (1000 data sets) of the multiple alignments tested the statistical robustness of the trees, with percentage values above 75% indicated by an asterisk. s/nt, substitutions per nucleotide. (**E**) Classification of the seven SARS-CoV-2 whole-genome sequences using ^a^ Nextstrain (https://nextstrain.org/ncov/, accessed on 30 April 2020), ^b^ PANGO Lineages (https://cov-lineages.org/index.html, accessed on 30 April 2020), and ^c^ GISAID database (https://www.gisaid.org/, accessed on 30 April 2020).

All seven C1 viral stocks (SARS-CoV-2 isolates), as well as the seven original NP clinical specimens, were first sequenced using a metagenomics approach by randomly constructing cDNA from extracted RNA, then sequencing on the MiSeq platform (Illumina). A total of 52,866,242 reads passing filter were obtained from the single MiSeq v3 600 cycle run, with 1426 cluster density, 0.63% error rate, 83.1% clusters passing filter, and 62% QScore ≥ Q30, with a good distribution of % reads passing filter across all 14 samples (range 3.32 to 12.1%, coefficient of variation of 0.31%). Reads were mapped and whole genomes assembled de novo or using a reference template (SARS-CoV-2 isolate Wuhan-Hu-1 NC_045512) depending on the software tool used. As expected, all four analyses (GISAID, DRAGEN Bio-IT Platform, Genome Detective Virus Tool, and CZ ID) generated similar results for all 14 samples. Although the total mapped reads (median 67,341 vs. 1,389,553 reads, *p* < 0.0001), median coverage depth (median 294 vs. 6519 reads, *p* < 0.0001), and length of SARS-CoV-2 genome analyzed (median 29,715 vs. 29,866, *p* < 0.006) were lower in the patient-derived samples compared to the SARS-CoV-2 isolates, the deep sequencing metrics allowed for the accurate analysis of all 14 whole-genome sequences (Figure 4A).

An initial phylogenetic analysis was performed to verify the identity and source of each SARS-CoV-2 isolate. As expected, the whole-genome sequences of the SARS-CoV-2 isolates were a perfect match to the corresponding patient-derived SARS-CoV-2 sequences, separated in phylogenetic clusters supported by 100% bootstrap values (Figure 4B). To characterize the first SARS-CoV-2 isolates from New Zealand, their phylogeny was reconstructed using 28 SARS-like betacoronavirus sequences downloaded from the GISAID database (https://www.gisaid.org/, accessed on 30 April 2020), showing that all seven viral sequences clustered with other SARS-CoV-2 sequences (Figure 4C). Further phylogenetic analysis using 70 contemporary SARS-CoV-2 sequences from different lineages downloaded from the GISAID database (https://www.gisaid.org/, accessed on 30 April 2020) (Figure 4D), as well as blast comparisons using a series of databases, i.e., Nextstrain (https://nextstrain.org/ncov/, accessed on 30 April 2020), PANGO Lineages (https://cov-lineages.org/index.html, accessed on 30 April 2020), and COVID-19 Research at UCSC (https://hgwdev.gi.ucsc.edu/covid19.html, accessed on 30 April 2020), classified the sequences of the first New Zealand SARS-CoV-2 isolates as Nextstrain Clade 19A, Pango Lineage B.55, and GISAID clade L for NZ1; Nextstrain Clade 19A, Pango Lineage B.31, and GISAID clade V for NZ2; Nextstrain Clade 20A, Pango Lineage B.1, and GISAID clade G for NZ3; Nextstrain Clade 20C, Pango Lineage B.1.369, and GISAID clade GH for NZ4; Nextstrain Clade 20C, Pango Lineage B.1, and GISAID clade GH for NZ5; Nextstrain Clade 19A, Pango Lineage B.31, and GISAID clade V for NZ6; and Nextstrain Clade 20A, Pango Lineage B.2, and GISAID clade G for NZ7 (Figure 4E).

Whole-genome sequences from the patient-derived samples and SARS-CoV-2 isolates, originally obtained with the MiSeq (Illumina) platform, were confirmed using the Ion AmpliSeq™ SARS-CoV-2 Research Assay in the Ion PGM™ System (Ion Torrent™, Thermo Fisher Scientific). The 14 samples were pooled and sequenced in three Ion 318™ v2 Chips (Thermo Fisher Scientific), with a median loading efficiency of 83% (range 81 to 85%), generating a median total of 5,207,910 quality reads (range 4,725,375 to 5,310,500 reads), and a median read length of 221 bp (range 215 to 224 bp). Reads were mapped and whole genomes assembled using the SARS-CoV-2 Research Plug-in Package (Thermo Fisher Scientific). Unlike the metagenomics approach based on the MiSeq platform (Illumina), no differences were observed between the sequencing metrics obtained with the amplicon-based Ion PGM™ System when comparing the clinical samples and SARS-CoV-2 isolates, i.e., total mapped reads (median 1,090,402 vs. 1,017,761 reads, *p* = 0.71), percentage of reads on target (median 99.96% vs. 99.97%, *p* = 0.83), mean coverage depth (median 6912 vs. 6671, *p* = 0.72), and the length of the SARS-CoV-2 genome analyzed (median 29,841 vs. 29,870 bp, *p* = 0.32), respectively. However, and more importantly, the whole-genome sequences from the patient-derived samples and SARS-CoV-2 isolates obtained with the Ion AmpliSeq™ SARS-CoV-2 Research Assay/Ion PGM™ System were a perfect match to the viral sequences obtained with the metagenomics/MiSeq approach (data not shown).

**Figure 5:**
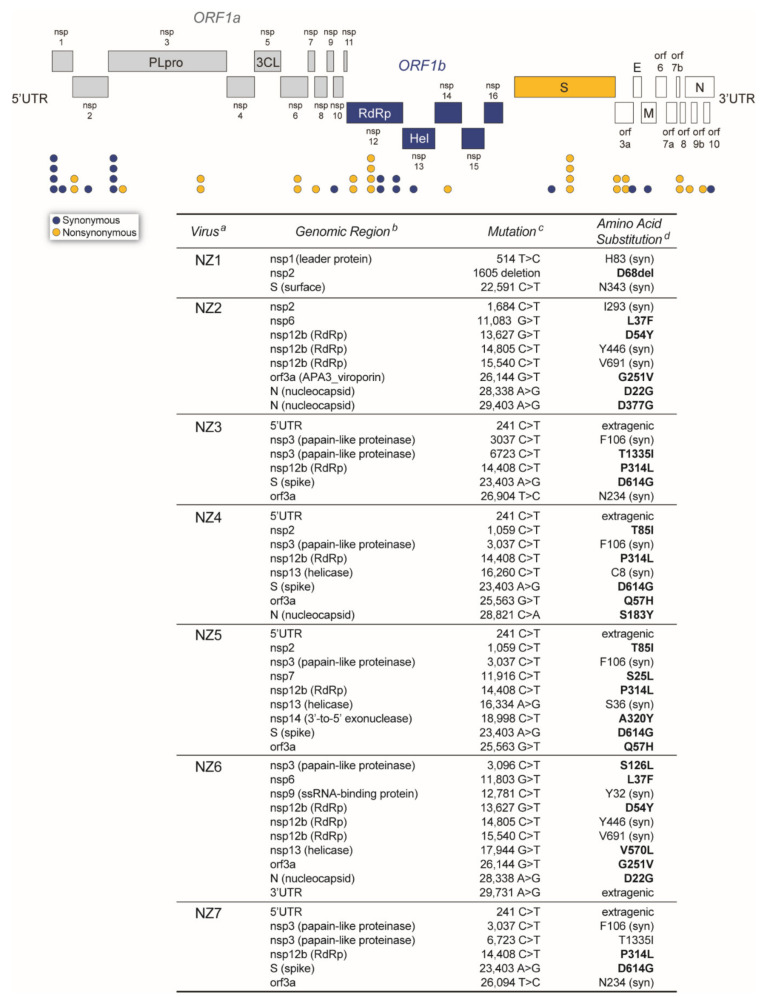
SARS-CoV-2 genome structure depicting the single nucleotide polymorphisms (mutations) detected in all seven SARS-CoV-2 isolates from New Zealand, relative to the Wuhan-Hu-1 (NC_045512) SARS-CoV-2 reference strain. ^a^ Source (patient) of the SARS-CoV-2 isolate. ^b^ SARS-CoV-2 genomic region. ^c^ Mutations identified with DRAGEN Bio-IT Platform (Illumina), Genome Detective Virus Tool (https://www.genomedetective.com/ accessed on 2 June 2020) [29], and CZ ID (https://czid.org/ accessed on 2 June 2020) [30]. ^d^ Amino acid substitutions annotated using Coronapp (http://giorgilab.unibo.it/coronannotator/ accessed on 2 June 2020) [31] are in bold. syn, synonymous mutation.

Finally, the seven SARS-CoV-2 isolates carried a variety of single nucleotide polymorphisms (SNP) relative to the reference sequence (SARS-CoV-2 isolate Wuhan-Hu-1 NC_045512), including a total of 27 non-synonymous mutations across the genome in different non-structural proteins (nsp), mainly in the RdRp (D54Y and P314L), the papain-like proteinase (nsp3, S126L and T1335I), and nucleocapsid (D22G, S183Y, and D377G) genes (Figure 5). Perhaps the most relevant amino acid substitution, i.e., the D614G in the spike gene, was observed in the sequence of four out of seven SARS-CoV-2 isolates (NZ3, NZ4, NZ5, and NZ7; Figure 5).

### 3.5. Microbiota of Patient-Derived Samples and SARS-CoV-2 Isolates

**Figure 6:**
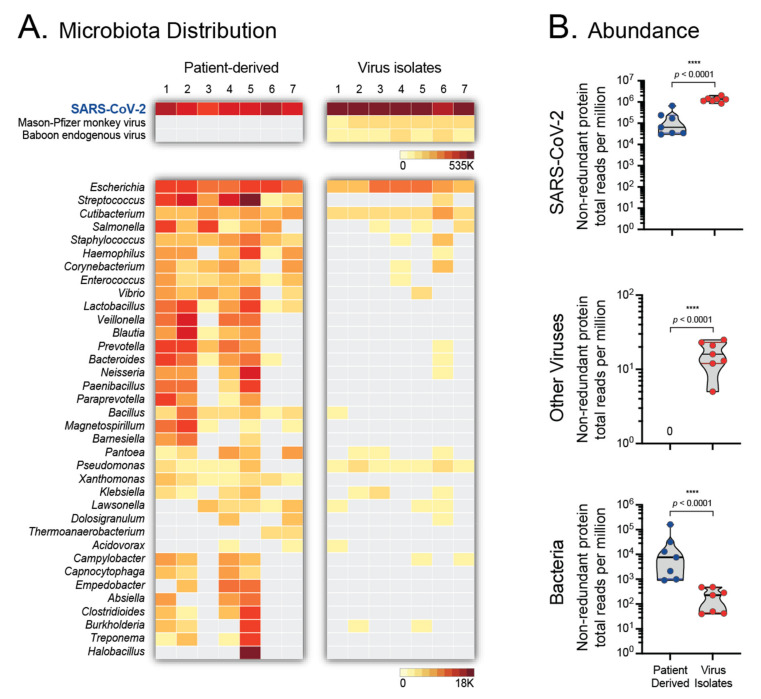
Microbiota analysis of patient-derived nasopharyngeal samples and SARS-CoV-2 isolates. Sequences obtained with the MiSeq (Illumina) platform were analyzed with CZ ID (https://czid.org/ accessed on 2 June 2020) to detect and quantify bacteria and viruses. (**A**) Taxon heatmaps represent the number of viral (top) and bacterial (bottom) sequences identified in both sets of samples. Full viral names are included, while bacteria are listed as genera. Heatmap scales in thousands (K) of total non-redundant protein reads per million are included. (**B**) Comparison of the abundance of SARS-CoV-2, other viruses, and bacteria sequences, quantified as total non-redundant protein reads per million, between the patient-derived nasopharyngeal samples (Patient Derived) and SARS-CoV-2 isolates (Virus Isolates). Wilcoxon–Mann–Whitney test was used to compare the abundance between both sets of samples. **** *p* < 0.0001.

The shotgun metagenomics sequencing approach using the MiSeq platform (Illumina) allowed for the analysis of the microbiota from both the patient-derived samples and the SARS-CoV-2 isolates, using CZ ID (https://czid.org/ accessed on 2 June 2020) [30]. Not surprisingly, the predominant viral sequences identified in all 14 samples corresponded to SARS-CoV-2 (Figure 6A), with a higher number of total non-redundant protein reads per million (NR_rpm) in the SARS-CoV-2 isolates compared to the clinical samples (median 1,318,105 vs. 64,443 NR_rpm, *p* < 0.0001, Figure 6B). Interestingly, a small number of reads associated with Mason-Pfizer monkey virus (median 11, range 3 to 15 NR_rpm) or Baboon endogenous virus (median 4, range 2 to 12 NR_rpm) were detected only in the SARS-CoV-2 isolates and not in the patient-derived samples (Figure 6). As expected, sequences corresponding to a number of bacterial genera were identified in the nasopharyngeal samples, including *Escherichia*, *Streptococcus*, *Staphylococcus*, *Veillonella*, *Prevotella*, *Bacteroides*, etc. (Figure 6). On the other hand, a limited number of bacterial sequences were detected in the SARS-CoV-2 isolates, mainly *Escherichia* and *Cutibacterium*, compared to the patient-derived samples (median 229 vs. 31,016 NR_rpm, *p* < 0.0001, Figure 6).

### 3.6. SARS-CoV-2 Growth Kinetics

**Figure 7:**
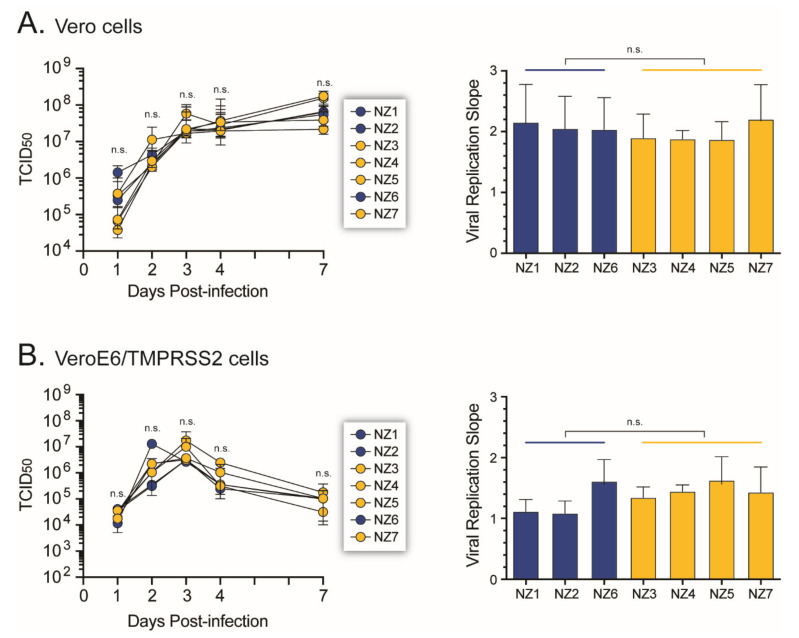
Viral growth kinetics analysis. The ability of the SARS-CoV-2 isolates to replicate in Vero (**A**) or VeroE6/TMPRSS2 (**B**) was quantified by determining TCID_50_ values by CPE, RT-qPCR assay [22], or using a cell protection assay (Pierce™ BCA Protein Assay Kit, Thermo Fisher Scientific). Viral replication slopes were calculated using the slopes between the TCID50 values at days 0 and 1, 0 and 2, and 0 and 3, corresponding to the exponential viral growth phase. All slope values were used to calculate the mean, standard deviation, and 10th and 90th percentiles. Differences in the mean values among all seven SARS-CoV-2 isolates and between viruses containing (yellow) or not (blue) the D614G substitution in the spike gene were evaluated using a one-way analysis of variance test or the Wilcoxon–Mann–Whitney test, respectively. n.s., not significant.

The ability of the SARS-CoV-2 isolates to replicate in Vero and VeroE6/TMPRSS2 cells was determined by measuring their replicative fitness in cell culture. All seven SARS-CoV-2 isolates showed similar growth kinetic profiles in Vero cells, with no significant difference in the viral replication slope, regardless of the presence or absence of the D614G amino acid substitution in the spike gene (Figure 7A). Similar results were obtained infecting the VeroE6/TMPRSS2 cells (Figure 7B), which are more susceptible to SARS-CoV-2 infection [41].

### 3.7. Susceptibility of SARS-CoV-2 Isolates to Antiviral Agents

**Figure 8:**
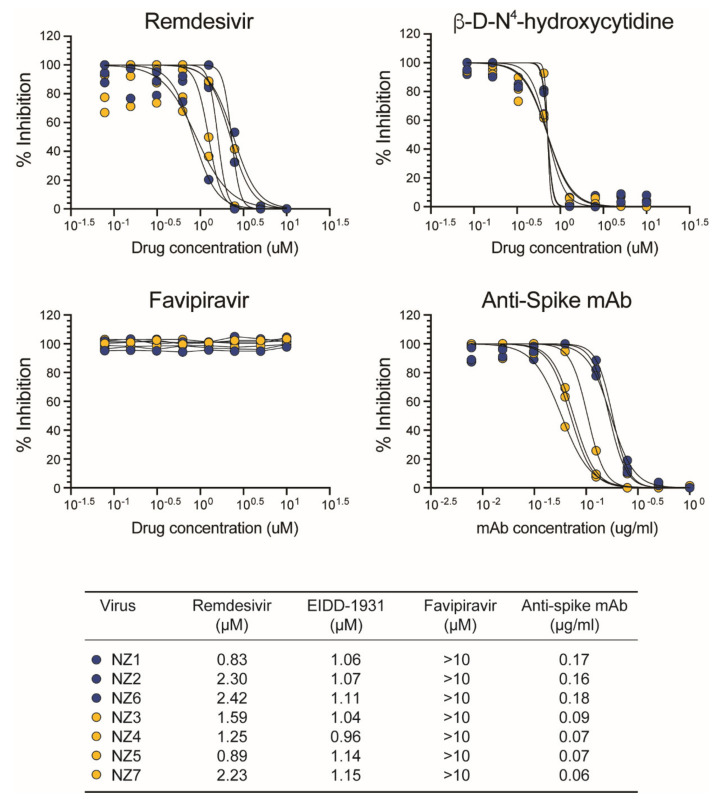
Susceptibility of SARS-CoV-2 isolates to remdesivir (GS-5734), β-D-N^4^-hydroxycytidine (NHC, EIDD-1931, molnupiravir), favipiravir (T-705), and a SARS-CoV-2 spike neutralizing monoclonal antibody was evaluated in VeroE6/TMPRSS2 cells. SARS-CoV-2 replication was quantified 72 h post-infection by CPE, RT-qPCR assay [22], or a cell protection assay based on the Pierce™ BCA Protein Assay Kit (Thermo Fisher Scientific). EC_50_ values are depicted in µM for remdesivir, β-D-N^4^-hydroxycytidine, and favipiravir or µg/mL for the SARS-CoV-2 spike neutralizing monoclonal antibody. SARS-CoV-2 isolates containing or not the D614G substitution in the spike gene are indicated in yellow or blue, respectively.

The ability of the SARS-CoV-2 isolates to replicate in the presence of three compounds that target viral RNA replication (remdesivir, β-D-N^4^-hydroxycytidine, and favipiravir), or a SARS-CoV-2 spike neutralizing mAb blocking viral entry, was assessed in VeroE6/TMPRSS2 cells. As expected, all viruses were susceptible to remdesivir (median effective concentration (EC_50_) of 1.59 µM, range 0.83 to 2.42 µM) and β-D-N^4^-hydroxycytidine (median EC_50_ of 1.07 µM, range 0.96 to 1.15 µM); however, up to 10 µM of favipiravir failed to exhibit significant activity against SARS-CoV-2 (Figure 8). Interestingly, although the SARS-CoV-2 spike neutralizing mAb was able to block the replication of all seven SARS-CoV-2 isolates, viruses carrying the D614G mutation in the spike gene (NZ3, NZ4, NZ5, and NZ7) were 2.4-fold more susceptible to the mAb than the wild-type viruses (median EC_50_ of 0.07 and 0.17 µg/mL, *p* < 0.0001, respectively; Figure 8).

## 4. Discussion

The 2009 swine flu pandemic, caused by the H1N1 influenza A virus, lasted approximately 14 months and was responsible for over 280,000 deaths [42]. SARS-CoV-2 arrived in late 2019 [1,8] and quickly became the second but so far major pandemic of the 21st century [9]. Since the days of the H1N1 influenza virus A virus, responsible for the 1918 Spanish flu [43], no other respiratory virus has been able to have such consequences on global health and the world economy like this new coronavirus [44]. The isolation and identification of SARS-CoV-2 as the viral agent responsible for the initial cases of acute respiratory illness in Wuhan, China [1], now called COVID-19, allowed for a massive worldwide response to try to control the spread of this new deadly virus [9]. Here, we describe the first isolation and characterization of SARS-CoV-2 in New Zealand, during the initial viral outbreak in March 2020, an effort that paved the way to a multitude of research projects in the country, including the development and validation of diagnostic assays, antiviral strategies, and a national COVID-19 vaccine program.

Following the initial isolation of the original Wuhan-Hu-1 SARS-CoV-2 in China [1], the virus was quickly identified and isolated from patient-derived samples in many different countries, e.g., Australia [39], South Korea [45], Hong Kong [46], USA [47], Italy [48], Finland [49], etc., with similar results: a virus capable of replicating in the original Vero cell line or any of its derivatives (i.e., Vero CCL-81, Vero 76, Vero E6, or VeroE6/TMPRSS2 cells), with virions showing a typical coronavirus structure [1,39]. Here, we used nasopharyngeal samples obtained from patients positive for SARS-CoV-2 infection in New Zealand’s South Island in early March 2020 [22]. Similar to other studies [39,45,46,47,48,49], we were able to isolate the virus from individuals with relatively high viral loads, evidenced by Ct values below 23 in the RT-qPCR test [22]. All seven virus isolates replicated and caused CPE in Vero cells, and were identified as SARS-CoV-2 by detecting the SARS-CoV-2 envelope gene, nucleocapsid protein, and spike glycoprotein using RT-qPCR, Western blot, and indirect immunofluorescence assays, respectively.

The initial identification of the virus isolates as SARS-CoV-2 was verified by electron microscopy, which has been widely used for the characterization not only of SARS-CoV-2 [1,39,45,50,51] but other species of coronaviruses [52,53,54,55]. We observed in all seven samples newly formed coronavirus particles being released from infected cells, with the fusion of vesicles releasing virions into the extracellular space as previously described [51]. We used electron tomography to inspect the spatial context of viral replication, virion assembly, and viral exit. Our data confirmed the major cellular remodeling events occurring during coronavirus infection [50,55,56], i.e., the abundance of double-membrane vesicles in the cytoplasm, important in viral RNA synthesis [52]. Viral assembly occurs in membranes associated with the endoplasmic reticulum and Golgi, with virions acquiring their membrane by budding inside the cisternae, forming particles averaging 80 nm in diameter [51,53]. These enlarged vesicles containing either single viral particles or groups of virions migrate towards the cellular periphery and fuse with the plasma membrane releasing the virus into the extracellular space [53]. The release of new virions is often associated with regions of the cell displaying filopodia-like protrusions. The frequency and shape of these structures in SARS-CoV-2-infected Vero cells suggest that these protrusions could be important for the egress and cell-to-cell spread of viral particles within epithelial monolayers [57].

The identity of the seven viruses isolated from the patient-derived samples was finally established by sequencing the entire viral genome. As of 6 January 2022, close to 6.8 million SARS-CoV-2 whole-genome sequences had been submitted to GISAID (https://www.gisaid.org/ accessed on 2 June 2020) [10]. This vast amount of information has been used to understand the evolution and epidemiology of the virus [8,58,59], leading to the classification of SARS-CoV-2 genetic diversity into clades or lineages [12]. Nine larger clades have been described in GISAID, 19 clades by Nextstrain (https://nextstrain.org/ncov/ accessed on 2 June 2020), while an increasing number of PANGO lineages (https://cov-lineages.org accessed on 2 June 2020) are used to track the transmission and spread of SARS-CoV-2 worldwide, including several variants of interest (VOI) or concern (VOC) [60]. Here, we first used a phylogenetic analysis to show that each one of the viruses was associated with their respective clinical sample, confirming the human origin of the New Zealand SARS-CoV-2 isolates. No other viral sequence was detected in the patient-derived samples; however, a small number of sequencing reads associated with Mason-Pfizer monkey virus (formerly Simian retrovirus) or Baboon endogenous virus were observed in the samples from the SARS-CoV-2 isolates. Interestingly, the complete genomes of these two retroviruses have been shown to be inserted into the Vero cell line genome [61]; therefore, the Vero cells used to isolate SARS-CoV-2 were the most likely source of the limited number of retroviral sequences.

All seven SARS-CoV-2 isolates were obtained very early in the pandemic, during the COVID-19 outbreak in New Zealand. Using phylogenetic analyses and different software tools available both online and in our laboratory, the whole-genome SARS-CoV-2 sequences were classified as B.55 (NZ1), B.31 (NZ2 and NZ6), B.1 (NZ3, NZ5 and NZ7), and B.1.369 (NZ4) based on the Pango Lineage nomenclature (https://cov-lineages.org/, accessed on 6 January 2022). SARS-CoV-2 sequences from the Pango lineage B.55 were predominant in Europe (https://cov-lineages.org, accessed on 6 January 2022) and patient NZ1 had returned from Germany before being diagnosed with COVID-19 [22]. Pango lineage B.31 SARS-CoV-2 sequences were more prevalent in the United Kingdom, New Zealand, and Australia, B.1 in the USA and United Kingdom, and B.1.369 in the USA and New Zealand (https://cov-lineages.org, accessed on 6 January 2022), suggesting that viruses infecting patients NZ2 to NZ7 may have been introduced from any of these countries. A study of more than 600 SARS-CoV-2 genomes obtained from patient-derived samples taken between February and May 2020 in New Zealand showed a high degree of viral diversity, including multiple A and B lineages, mostly B.1, B.1.1. and B.1.26, with a low frequency of B.31 [62]. Worldwide cumulative prevalence of B.55, B.31, and B.1.369 SARS-CoV-2 Pango lineages has diminished to less than 0.5%, with sequences from the B.1 lineage lingering at 2% (https://outbreak.info/, accessed on 6 January 2022). In New Zealand, SARS-CoV-2 sequences from lineages B.55, B.31, and B.1.369 have a cumulative prevalence of <0.5%, 1%, and 1%, respectively, although they were last detected in the country in April 2020. On the other hand, SARS-CoV-2 B.1 sequences still account for 2% of the cumulative cases in New Zealand (https://outbreak.info/, accessed on 6 January 2022).

SARS-CoV-2 continues to evolve, adapting to replicate in the new human host [5,7,9,12,13,59]. Despite the 3′ to 5′ exonuclease proofreading ability provided by the nsp14 enzyme [63], the virus is accumulating a number of non-synonymous mutations (amino acid substitutions) across the entire genome, showing higher diversity in the spike and nucleocapsid genes (https://nextstrain.org/ncov/gisaid/global, accessed on 10 January 2022), which has led to the generation and selection of novel SARS-CoV-2 variants [9,13]. In this study, a median of eight mutations (range 3 to 10 mutations) were identified in the whole-genome sequences of the seven SARS-CoV-2 isolates, distributed along different open reading frames. A series of amino acid substitutions were identified, including some in the leader protein, papain-like proteinase, RdRp, viroporin, and nucleocapsid genes. Interestingly, while most of these non-synonymous mutations have not been associated with changes in viral phenotype, the D614G substitution in the spike gene was detected in four SARS-CoV-2 isolates (NZ3, NZ4, NZ5, and NZ7). Identified in February 2020, D614G was the first amino acid substitution to be associated with an increase in SARS-CoV-2 transmissibility [64,65], which allowed the D614G variant to reach close to 100% global prevalence by mid-2020 [13,64,66]. The D614G substitution was also present in the first VOC, Pango lineage B.1.1.7 (VOC Alpha), and has been present in all VOCs identified thus far, including B.1.617.2 (Delta) and B.1.1.529 (Omicron) (https://outbreak.info/, accessed on 6 January 2022).

SARS-CoV-2 variants containing the D614G substitution have been shown to have a slight increase in infectivity in Vero-E6, Vero-81, and Huh7 cells [64,65]; however, here, we showed that the D614G substitution does not confer a clear replicative advantage in Vero or VeroE6/TMPRSS2 cells. The enhanced infectivity and replication fitness of SARS-CoV-2 D614G seems to be more evident in primary human airway cells and the upper respiratory tract of animal models, e.g., hamsters [65,66]. On the other hand, all seven SARS-CoV-2 isolates were equally susceptible to remdesivir and β-D-N^4^-hydroxycytidine (molnupiravir), two drugs authorized by the U.S. Food and Drug Administration for the treatment of COVID-19 [67,68]. Interestingly, SARS-CoV-2 isolates with the D614G were more susceptible to neutralization by a commercial monoclonal antibody targeting the spike glycoprotein. This is similar to previous studies showing a slight increase in susceptibility to neutralization by mAbs and plasma from convalescent or vaccinated individuals [69,70], although other studies have shown an increase in resistance to neutralization in viruses carrying the D614G substitution [65,71].

## 5. Conclusions

We first isolated SARS-CoV-2 in New Zealand almost two years ago, in early 2020. We were able to fully characterize these virus isolates, including their growth kinetics in cell culture, as well as visualize them by electron microscopy. We analyzed their whole-genome sequences using two different but complementary deep sequencing platforms, which allowed for the classification in clades/lineages and the identification of key amino acid substitutions, such as the D614G, associated with significant phenotypic characteristics. Since then, we have isolated other SARS-CoV-2 variants that have reached our shores, such as B.1.617.2 (Delta), B.1.621 (Mu), and the highly transmissible B.1.1.529 (Omicron). Altogether, this seminal work allowed for early access to SARS-CoV-2 isolates in New Zealand, clearing the way to numerous clinical and scientific research projects in the country, including the development of a national COVID-19 vaccine development program.

## Data Availability

Raw data can be requested by contacting the corresponding authors.

## Supplementary Materials

The following are available online at https://www.mdpi.com/article/10.3390/v14020366/s1, Figures S1–S3: Electron microscopy images of SARS-CoV-2-infected Vero cells.
