# Supplementary material for: Characterization of the First SARS-CoV-2 Isolates from Aotearoa New Zealand as Part of a Rapid Response to the COVID-19 Pandemic
Source: Viruses. 2022 Feb 10;14(2):366. doi: 10.3390/v14020366 (PMC8877023; doi:10.3390/v14020366)
Supplement: Supplementary file 1 [file viruses-14-00366-s001.zip › viruses-1584175-supplementary.pdf]

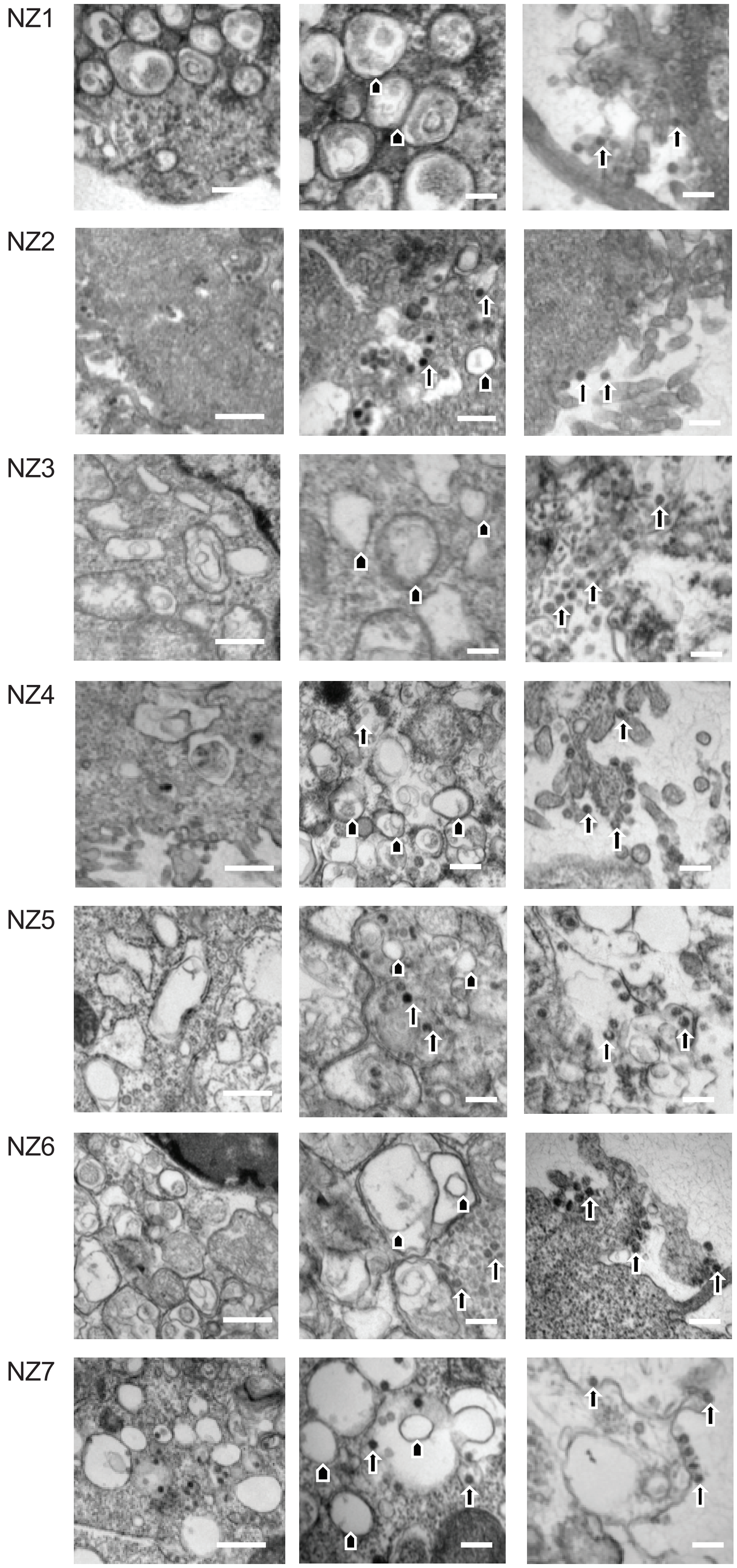

Figure S1. Electron microscopy of Vero cells infected with the different SARS-CoV-2 isolates (NZ1 to NZ7). Panels show areas of cytoplasm of infected cells (left), double membrane vesicles and viral cells located inside the cells (centre), and sheded virions (right). Virions are indicated with arrows and double double membrane vesicles with arrowheads; scale bars 1 μm (left), 500 nm (center and right).

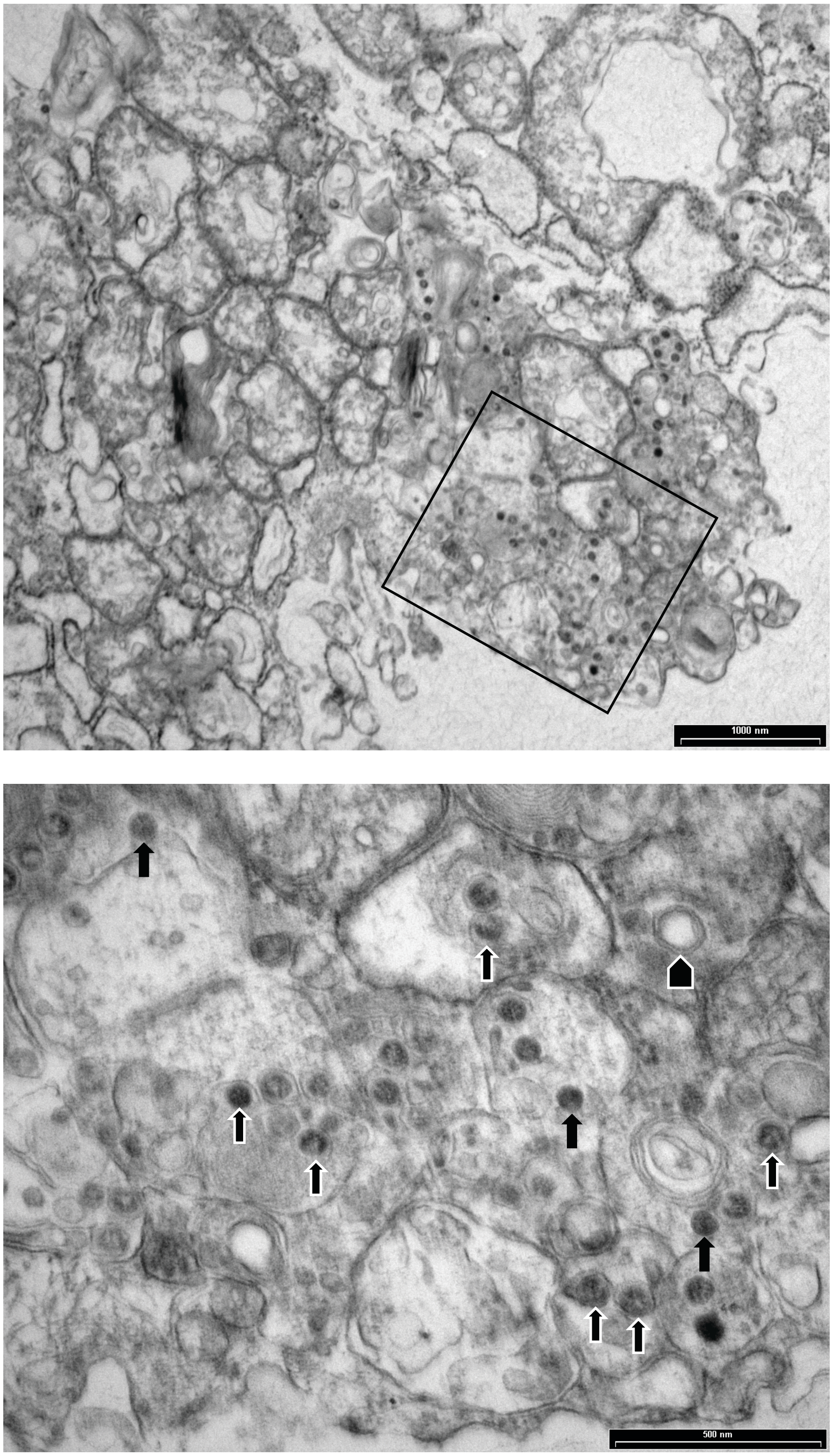

Figure S2. Electron microscopy of Vero cells infected with SARS-CoV-2. Image of an assembly zone located at the periphery of the infected cell is shown in the top panel. Enlarged area (also shown in the bottom panel) indicates a region displaying double membrane vesicles (arrowheads), virions (black arrows), and wrapped virions (white lined arrows).

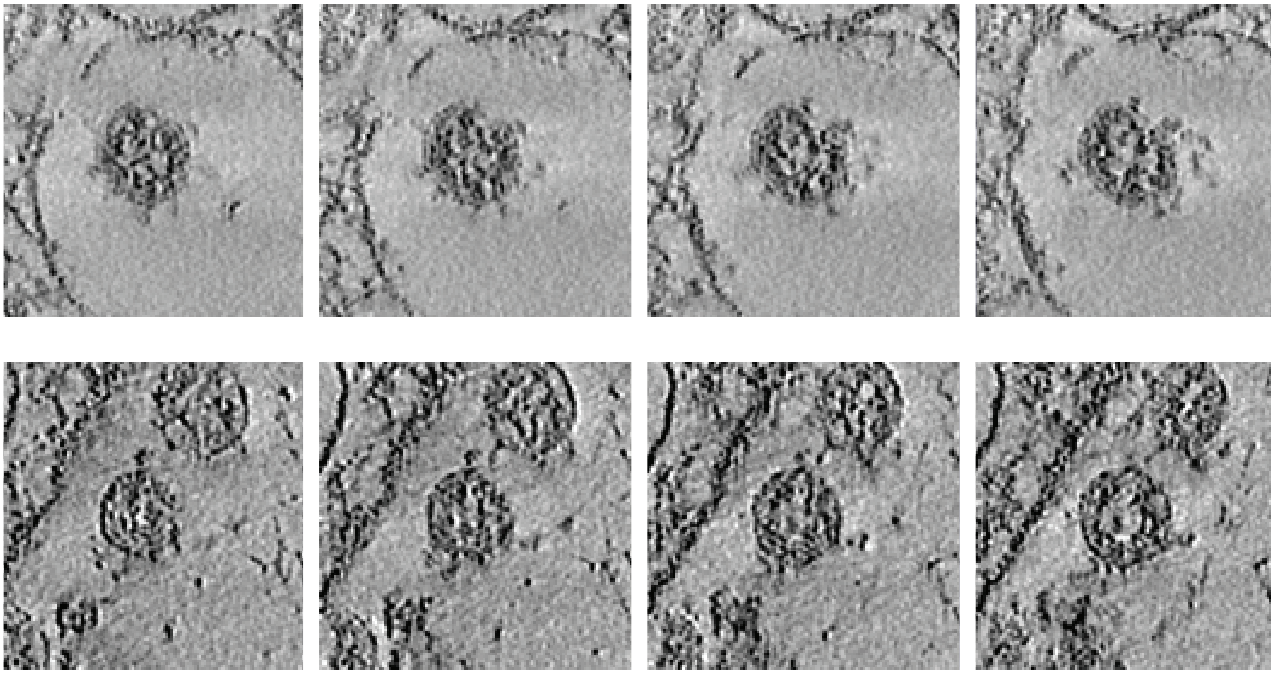

Figure S3. Electron tomography of SARS-CoV-2. Successive sections 10 nm thick through tomographic reconstruction of virions present inside the cell (top panels) and extracellular area (bottom panels). The diameter of a virion is approximately 80 nm.
